# Barriers to healthcare data quality and recommendations in public health facilities in Dire Dawa city administration, eastern Ethiopia: a qualitative study

**DOI:** 10.3389/fdgth.2024.1261031

**Published:** 2024-03-14

**Authors:** Abebe Tolera, Dawit Firdisa, Hirbo Shore Roba, Aboma Motuma, Monas Kitesa, Admas Abera Abaerei

**Affiliations:** ^1^ School of Public Health, College of Health and Medical Sciences, Haramaya University, Harar, Ethiopia; ^2^ School of Nursing and Midwifery, College of Health and Medical Sciences, Haramaya University, Harar, Ethiopia; ^3^ School of Pharmacy, College of Health and Medical Sciences, Haramaya University, Harar, Ethiopia

**Keywords:** healthcare data quality, barriers to data quality, health information, qualitative study, inductive analysis, semantic analysis

## Abstract

**Background:**

Maintaining good quality of healthcare data at various levels is a critical challenge in developing countries. The barriers to healthcare data quality remain largely unexplored in eastern Ethiopia.

**Objective:**

This study aimed to assess the barriers to quality of healthcare data in urban public health facilities in the Dire Dawa city administration from 7 April to 7 May 2019.

**Methods:**

An institutional-based qualitative exploratory approach was used among 17 purposefully selected key informants. In-depth interviews were inductively coded using the ATLAS.ti 7.5.4 version software. Inductive analysis was used by semantically analyzing the explicit content of the data to determine our themes.

**Results:**

Several key themes and subthemes with different barriers, some of which are mutually non-exclusive, were identified. These include: ***Organizational Barriers:*** Lack of an adequate health management information system and data clerk staff, poor management commitment, lack of post-training follow-up, work overload, frequent duty rotation, lack of incentives for good performers, lack of targeted feedback, and poor culture of information use. ***Behavioral/Individual Barriers:*** Gaps in the skill of managers and health professionals, lack of adequate awareness of each indicator and its definitions, inadequate educational competence, lack of feeling of ownership**,** poor commitment, lack of daily tallying, and lack of value for data. ***Technical Barriers:*** Lack of a standard form, diverse and too many data entry formats, manual data collection, shortage of supplies, failure to repair system break down in a timely manner, interruption in electricity and network, delay in digitizing health information systems, lack of post-training follow-up, and inadequate supervision. ***External Barriers:*** Poor collaboration between stakeholders, dependence on the software program of non-governmental organizations, and very hot weather conditions.

**Conclusion:**

Diverse and complex barriers to maintenance of data quality were identified. Developing standardized health management information system implementation plans, providing advanced supervisory-level training, supportive supervision, and site-level mentorship may be very effective in identifying and resolving bottleneck data quality issues. Healthcare managers should understand the imperative of data quality and accept responsibility for its improvement and maintenance. Interventions targeted only at supplies will not fully overcome limitations to data quality. Motivation of staff and recognition of best performance can motivate others and can create cooperation among staff.

## Introduction

Maintaining and obtaining good-quality healthcare data is critical for improved healthcare delivery to the population (1–3). Good routine healthcare data quality is important for continuity of care, good clinical practice, program management, planning, resource allocation, and policy decisions that are crucial for patient care (3–6). Furthermore, routinely collected health data (RCD) with good quality and better feasibility can be used to expand the research agenda and offer new design and data collection options (7).

Despite this, both developed and developing countries have limited and highly variable data accuracy, privacy, and security (8–10). Unlike developed nations, developing countries have reported having a large amount of unreliable health data, poorly motivated human resources, and a weak information technology infrastructure (11, 12). Poor quality of information in patient data may be a cause of poor quality of care and be associated with additional healthcare costs and productivity loss (13, 14).

In Ethiopia, the quality and utilization of health information remains weak. The incompleteness of healthcare data is the main problem, particularly in primary health facilities and district levels (11, 15). Several studies in Ethiopia showed that the quality of healthcare data is very low (4, 16). Studies among the health facilities of the Dire Dawa city administration and Addis Ababa showed that the overall quality of the data was 75.3% and 82.5%, respectively, which was below the national expectation level (11, 17). A gap in the level of knowledge and skill of health workers significantly influences data management processes, timeliness, completeness, and accuracy at the point of service delivery (5, 13, 17).

In the current Ethiopian health sector transformation plan, the information revolution aims to transform the culture of data utilization through cultural changes in the health information systems (HIS) and their digitalization and scaling (18, 19). To improve healthcare data quality, the Ethiopian Federal Ministry of Health (EFMOH) collaborated with several national and international organizations (5, 11). Despite this, the quality of healthcare data needed to get valid information to make decisions about health programs is weak or insufficient in Ethiopia (20). Several reports have shown variability in the quality of both indicators and data elements (20–22).

In particular, there is little evidence on the factors that affect the quality of healthcare data in low- and middle-income countries. The barriers to healthcare data quality remain largely unexplored in eastern Ethiopia. Therefore, this study aimed to identify the barriers to quality of healthcare data among public health facilities in the city administration of Dire Dawa.

## Methods

### Study setting and design

A qualitative exploratory phenomenological study was conducted in the public health facilities in Dire Dawa city administration in eastern Ethiopia from 7 April to 7 May 2019. Dire Dawa city is divided administratively into two *woredas*: the city proper and the non-urban *woreda*, or Gurgura. There were 15 health centers (eight urban and seven rural), 2 hospitals, and 32 health posts under the city administration. These public health facilities served a population of 480,000 in reproductive, maternal, neonatal, child and adolescent health, major communicable diseases, non-communicable diseases, surgical and injury care, emergency and critical care, neglected tropical diseases, hygiene and environmental health services, health education and behavior change communication services, and multisectoral nutrition interventions.

### Study population and sampling approach

A purposeful sampling was used to select 17 key informants to improve the understanding of information-rich cases on barriers to healthcare data quality. A total of 12 key informants from the health management information system (HMIS), 1 regional HMIS focal person, 2 hospital heads, and 2 health center heads working in urban public health facilities were interviewed.

### Data collection and quality control procedures

Data were collected for one month during working hours each day. The key informants were pre-identified and scheduled for the interview. Two trained epidemiologists experienced with qualitative data collection conducted the in-depth interviews after 2 days of training. Three-item questions with nine sub-questions and in-depth probing were used for the in-depth interviews, which were adopted and contextualized for this study purpose from other studies (23, 24).

The key informants who were unavailable at the time of the study were repeatedly visited to minimize high non-responses. A Sony ICD PX470 sound recorder was used to record the responses of the key informants. Upon completion of each in-depth interview, a trained language professional produced a complete transcript and translation of the data for data entry and analysis since the data were collected in local languages (Afan Oromo and Amharic). The rigor of the qualitative data was ensured through thoughtful and deliberate planning, ongoing application of researcher reflexivity, and honest communication between the researcher and the audience about the study and its results.

### Operational definition

• **Healthcare data:** the healthcare data considered in this study included electronic health records (including electronic medical record (EMR) and paper based health data record (PHR)), administrative data, patient and disease registries, and health surveys.
• **The quality of the data** is described in three dimensions: precision, completeness, and timeliness (25).
• **Barriers to healthcare data quality:** the obstacles that can affect the timeliness, accuracy, completeness, accessibility, and use of data from an HIS.
• **Data clerk:** Someone who transfers data from paper formats into computer files or database systems.
• **Health information technicians (HIT):** a professional who develops, maintains, and implements health records processing, storage, and retrieval systems in medical facilities and other healthcare settings to meet the legal, professional, ethical, and administrative records-keeping requirements of health service delivery.
• **Health posts** are primary levels of care that aim to promote communities’ abilities to improve their own health services. The main data collected at this level are the community health information system (CHIS and/or eCHIS).
• **Phenomenological exploratory qualitative study** is a qualitative research approach that seeks to understand the essence of a particular phenomenon through a detailed exploration of individual experiences such as emotions, perceptions, and awareness.
• **Research reflexivity** is about acknowledging our role in the research. As qualitative researchers, the researchers are part of the research process, and their prior experiences, assumptions, and beliefs will influence the research process.
• **Inductive coding** is a ground-up approach where we derive our codes from the data and where we allow the narrative or theory to emerge from the raw data itself.
• **Inductive analysis**, as one approach to qualitative content analysis, involves collecting and analyzing data without preconceived categories or theories.
• **Semantic analysis** is the process of drawing meaning from text.
• **Explicit content** are data that are transparent and easy to identify.
• **Timely and targeted feedbacks** are feedbacks given as soon as possible specific to the identified (targeted) event, action, or behavior that needs to be addressed.
• **Standardization of data quality** is the act of using consistent methods to collect data with the consideration of key stakeholders.

### Data analysis approach

The sociodemographic data of the study participants were summarized using a simple frequency table and median. The recorded versions of Amharic and Afan Oromo were transcribed and translated back to the English version. The ATLAS.ti 7.5.4 version software was used for data analysis. Coding was based on inductive coding, and inductive analysis was used by semantically analyzing the explicit content of the data to determine our themes. Line-by-line examination of each sentence served as the unit of analysis for coding purposes, and the participants’ own words guided the development of the quotations.

## Results

### Sociodemographic characteristics of the study participants

A total of 17 key informants (12 HMIS/HIT staff, 2 hospital heads, 2 health center heads, and 1 regional HMIS focal person) were involved in these in-depth interviews. The median age of the study participants was 33 [±2 interquartile range (IQR)] years. In total, 10 of the key informants were women (58.82%) and 10 of them lived in an urban setting (Table 1), 9 of them were married (52.94%), and 8 (47.06%) of them were holders of diplomas (Table 1).

**Table 1:** Sociodemographic characteristics of study participants, Dire Dawa, eastern Ethiopia, 2019.

Variable	Category	Frequency	Percentage
Sex	Female	10	58.82
	Male	7	41.28
Residence	Urban	10	58.82
	Rural	7	41.28
Educational level	Diploma	8	47.06
	Degree	5	29.41
	Masters and above	4	23.53
Work experience	<7	10	58.82
	>7	7	41.28
Profession	HIT	12	70.59
	Nurse	2	11.76
	Health Officer	2	11.76
	HMIS focal person	1	5.88

### Identified barriers to health data quality

Multiple, some of which are mutually non-exclusive, barriers to data quality were cited by key informants during in-depth interviews. Common themes were organized as organizational, technical, behavioral, and environmental barriers during the analysis (Table 2). The key themes and subthemes with identified barriers are presented in Table 2.

1. Organizational barriers

**Table 2:** Key themes and subthemes with identified barriers to health data quality, eastern Ethiopia, 2019.

Themes	Subthemes	Identified barriers
Organizational barriers	Organizational structure and policies	• Lack of adequate HMIS and Data clerk staff • Lack of accountability • Poor management commitment
	Tasks	• Poor coordination • Lack of post-training follow-up • Being new staff • Work overload • Shortage of data entry formats and delays in supply)^b^ • Frequent duty rotation
	Incentives	• Poor work motivation and recognition • Lack of incentives for good performers
	Information and decision processes	• Lack of targeted feedback • Poor culture of information use^a^ • Request for report on the weekend
Behavioral/individual barriers	Insufficient skills in data use core competencies	• Gap in skill of manages • Lack of awareness of new disease classification • Lack of adequate skill by health professionals • Lack of adequate awareness of each indicator and its definitions • Inadequate educational competence
	Poor individual commitment and motivation	• Lack of responsibility and accountability • Lack of feeling ownership • Poor commitment • Lack of daily tallying
	Poor attitude	• Lack of value for data^c^ • Reluctance and negligence^d^
Technical barriers	System design	• Lack of standard form • Diverse and too many data entry formants • Frequently changing data entry formants • Manual data collection
	Input issues	• Shortage of supplies • Failure to repair system break down in a timely manner • Lack of purchasing of accessories in a timely manner • Interruption in electricity and network • Delay in digitizing health information systems
	Inadequate technical support	• Lack of post-training follow-up • Poor support • Inadequate supervision
External barriers	Stakeholders’ roles	• Poor collaboration between stakeholders • Dependence on NGOs’ software program • Very hot weather conditions.

^a^ Poor information-use culture is a culture that is non-conducive to effective information management where the value and utility of information in achieving operational and strategic goals is unrecognized.

^b^ Delays in the supply of data entry formats mean an untimely or irregular supply.

^c^ Lack of value for data means misunderstanding the importance of healthcare data for service provision.

^d^ Reluctance and negligence mean an unwillingness to gather, fill, and repot healthcare data in a timely manner.

The inability to use data for decision-making, particularly at lower levels, and the lack of timely and targeted feedback (specific to identified gaps) was the main barrier cited by key informants to healthcare data quality. Insufficient motivation and recognition were also cited as a barrier to the quality of healthcare data by all HMIS focal persons and health center managers. A 34-year-old woman head of one of the health centers states that

*Lack of recognition of good staff can derail staff morale. Everyone may feel that there is no difference between hard-working and poorly working staff. As a result, hardworking staff become reluctant and lack interest. They say, “just write what you want and report anything. There is no value in reporting correct data or not.”*

Despite a report by most key informants on work overload as a major barrier to healthcare data quality, a 27-year-old male HIT from one of the health centers disagreed with work overload as a barrier to data quality.

A 33-year-old HIT from another health center states that frequent rotation of staff from one unit to another is one challenge to maintaining data quality. He said

If *one health provider working on family planning is assigned to the TB clinic, he will be annoyed about the new registers and forms. She will write data on wrong registries and forms. This is because he will be assigned to a new service delivery unit without adequate training on the new forms and registries. Everything becomes new to them.*

2. Behavioral/individual barriers

The main behavioral barrier stated by almost all key informants is the lack of responsibility and accountability of healthcare providers. A 34-year-old male key informant in one of the health centers said

There *is reluctance of healthcare professionals. They usually avoid the responsibility of tallying and filling in the data collection forms. Timeliness is affected by carelessness or referring one's duty to others. They forget when to list and fill the registers. This will increase the number of data to be filled next time. They then get frustrated and report unnecessary data. They also sometimes think that it is their teammate's responsibility. They always disagree on this issue.*

Another major behavioral barrier cited by almost all key informants is the inadequate knowledge of the healthcare providers of each health indicator and their definitions. They stated that most healthcare providers were not interested in reading and understanding the national classification of diseases and healthcare indicators. A 28-year-old female key informant in one of the health centers said

*The main data quality barrier is the lack of adequate awareness of each indicator and its definitions. Health professionals do not know the appropriate codes and data elements under each indicator. As a result, they (healthcare workers) usually submit incomplete and inaccurate data. They get agitated and fill out forms haphazardly. Then it is a dual burden for me to go and check its comparability between records and reports.*

Another issue raised by 12 key informants is the attitudes of health professionals toward the value of data. A 34-year-old male key informant in one of the health centers said

*We cannot solve this problem even if we hired a sufficient number of HIT personnel unless health professionals feel ownership of the information collected. Data are more useful to health professionals than to HIT professionals.*

3. Technical barriers

One of the major technical barriers to healthcare data quality is the lack of the necessary training for new personnel. A 36-year-old male head in one of the health centers said

*New staff members are assigned to the public service without adequate training and knowledge of the quality of data. They wait for us to inform them about how to count and register data*.

Similarly, a 32-year-old female key informant stated that

*The other problem that health professionals have raised is the diverse tally sheet and the change of these tally sheets from time to time. Different institutions (including NGOs) want different data reports. They usually bring different tally sheets. As a result, health professionals get confused and their morale fades to fill all these formats. In addition, the tally sheets are too many to fill, and they also get tired of filling all these formats.*

Failure to repair the system, replace it, and purchase accessories in a timely manner was also identified as a technical barrier. Many key HIT informants also cited a lack of adequate functional computers as one barrier. A 40-year-old male head in one of the hospitals said

*Some stakeholders also add some registrations for their own report in addition to those provided by the government. Now we are ordered to fill only those forms provided by the government, and any interested institution can obtain the type of data they want from these registries because it includes all data elements*.

The occasional running out of data recording materials was cited by most key informants as one main barrier to healthcare data quality. A 32-year-old female HMIS focal person at one of the referral hospitals stated

*First, the data is currently collected manually on hardcopy. As a result, there is shortage of supplies such as registers, tally sheets, and other forms. As these forms are not provided in a timely manner, it contributed to an incomplete report of the data elements and an overall incomplete medical record.*

4. External barriers

Some key informants mentioned some external barriers, including poor collaboration between stakeholders, dependence on NGOs’ software programs, and very hot weather conditions.

### Recommended measures to improve data quality

The key informants indicated different suggestions and recommendations. These include electronic data recording, the provision of adequate training, standardizing forms and registries, timely feedback, hiring appropriate IT professionals, improving the data-use culture, a clear job description, frequent mentoring, regular supervision and support, motivation and incentives, educational opportunities, and taking timely corrective measures. The most frequent recommendation stated by the key informants is to take timely and sometimes punitive measures against those staff who do not carry out their responsibilities. Adequate supervision and recognition of the best performers were recommended by almost all key informants.

A 28-year-old female HIT stated

*As I told you, recognition and incentives for staff can improve data quality. Training in data management should also be considered. Moreover, before I advise advise the concerned entities to provide adequate training on disease classification. Health professionals should also ask for help if they lack adequate knowledge and skills on how to maintain quality health data.*

A 32-year-old female HMIS focal person at one of the hospitals said

*Formats should be standardized and after training, mentoring should be there. Feedback should be provided in a timely manner. On-job follow-up and supervision should be there. Providing timely solutions to problems and questions of health professionals can improve all this.*

## Discussion

This study focused mainly on the identification of barriers to the quality of health data among public health facilities in the administrative city of Dire Dawa. A key finding from our study showed major barriers to healthcare data quality were classified as organizational, behavioral/individual, technical, and external barriers. This finding is consistent with previous studies (1, 21, 23, 26, 27), which showed barriers to high-quality health data arising mainly from the data generation process, particularly at the level of documentation by healthcare providers and data clerks.

Our study showed a shortage of data entry formats and/or delays in supplies affected healthcare data quality. This finding is consistent with studies from Canada (1), Iran (28), Kenya (29, 30), and Ethiopia (21), which indicated documentation of service data within the healthcare facility is perhaps the most important resource for data quality. Similarly, studies from Ethiopia (31) and 41 low-income countries (32) identified poor availability of resources and lack of performance feedback as the most frequent weaknesses in the information systems. These had an impact on staff motivation, timeliness, and completeness of the data.

Evidence-based planning and interventions are crucial to improving service delivery. This study identified a poor culture of information use, particularly at the lower level, which affects the quality of healthcare data. This negatively affects the commitment and value given to data by the healthcare providers. This finding is also consistent with studies from the Southern Nations, Nationalities, and People's Region (SNNPR) of Ethiopia (20, 31), Iran (28), Thailand (33), Canada (1), and Kenya (30). Health facility managers and other responsible bodies usually do not use the collected data owing to reliability and accuracy problems. Using and communicating accurate and timely information to the healthcare community, decision makers, and the public to effect behavior change and obtain resources and support for effective action” is beneficial in improving data quality and program implementation (34). Healthcare managers should understand the imperative of data quality and accept the responsibility for its improvement and maintenance. Another key finding from our study was that inadequate HMIS and HIT staffs were a major barrier to healthcare data quality. This finding is consistent with other studies from Ethiopia (1, 21), Canada (26), and Australia (35) in which insufficient human resources and deficiencies in the training of available human resources were identified to affect the quality of healthcare data. Insufficient human resources can, in turn, affect the workload that a given care provider and HMIS staff take to code and document service data. Despite the disagreement of some key informants, work overload was identified as affecting healthcare data quality. This finding is consistent with a study from China (36) and Canada (1) in which paper-based documentation and volumes of cases that physicians dealt with adversely affected data quality during coding (1). Workflow is a critical aspect of a healthcare system (37).

The lack of targeted feedback, post-training follow-up, and recognition of good performers, conversely, affected staff performance in managing and maintaining data quality. This finding is consistent with studies in Northwest Ethiopia (32, 38), Ethiopia (38), and Kenya (29). The absence of recognition and incentives for good performers impedes the feelings of ownership and value of the data of healthcare providers. A study by Ayele et al. (21) and Haftu et al. (22) showed that supportive supervision and mentorship are associated with data quality (22). Insufficient supervision may result in incomplete charts, which in turn affect data quality. Interventions targeted only at supplies will not fully overcome limitations to data quality. The motivation of staff and recognition of best performance can motivate others and can create cooperation among staff. User participation is one of the critical aspects of healthcare programs (37). Lack of ownership (responsibility and accountability) and lack of value for data were identified as factors that affect the quality of healthcare data. This study is consistent with studies from Canada (1), South Africa (22, 39), and Iran (28) where physicians played a major role in influencing the quality of administrative data. Building the capacity and control to promote values and beliefs among members of an organization for the collection, analysis, and use of information to accomplish its goals is crucial to good-quality health data (31, 40). A qualitative study in Canada showed a communication divide between coders and physicians, resulting in coders feeling blamed for quality issues around coded data (26). Unlike other healthcare providers, health information management professionals are pressured to meet the reporting timeline. This causes the other healthcare care providers to feel a lack of commitment to complete the patients’ cards (1).

User experience is a critical aspect of any program's success (37). Participants in this study cited lack of awareness of the new classification of diseases, inadequate educational competence, and inadequate technical skills as barriers from the side of healthcare providers. This finding is consistent with studies from Ethiopia (31), Canada (1), and Iran (28), Lucyk et al. (1) and Haftu et al. (22). This indicates that behavioral factors such as motivation, confidence, and demand for data, task competency, and problem-solving skills adversely affect chart documentation. Healthcare provides lack of knowledge and awareness on how to code and document patient service adversely influences data quality. Tang et al. (26) showed that the difference in the use of terminologies by physicians and coders to describe clinical diagnoses affects the production of high-quality administrative data (26). A study in the USA showed that training and audit procedures resulted in high-quality administrative data (41).

Improving the competence of the health information system task and the engagement of the caregiver with other staff can improve data quality.

In addition, diverse, too many, and frequently changing data entry formats were identified to affect healthcare data quality. Despite recent improvements, the participants in this study said many organizations, in addition to national data registries, requested to fill in the data in different and diverse formats. This in turn resulted in frustration of the healthcare workers with filling out all the forms and registries. This is sometimes complicated by a delay in analyzing the data due to a shortage of data entry formats or a delay in supplies. This finding is consistent with studies in different countries, which indicated a lack of comprehensive and standardized national reporting formats and stakeholder requirements for the use of very different tools impeded healthcare data quality (11, 20, 30, 31, 42). A lack of resources and standardization of a data set can directly hinder our efforts to improve healthcare delivery.

This study identified gaps in the computerization of healthcare delivery. Healthcare delivery and administrative data were collected manually or in hard copy. The study participants cited that there were no national standard EMRs. The facilities used to depend on an EMR software program supplied by an NGO. Despite enormous advances in computerized healthcare information systems in the previous two decades, low-income countries still lag behind other middle- and high-income countries in the digitalization of service delivery (30, 43). Studies in Kenya (44) and USA (45) showed that electronic systems such as EMR-based data recording have significant impacts on service data quality. Furthermore, the failure to repair system breakdowns and purchase accessories in a timely manner has complicated the data documentation process. Staffs get agitated and lose personal motivation when their organizations fail to recognize their effort, take advantage of all necessary inputs, and repair damaged equipment.

Unlike a Kenyan study (29), this study showed that poor collaboration of stakeholders with health facilities was one major barrier to maintaining data quality, particularly when using electronic systems for data recording and capacity building areas. This finding is consistent with studies in Iran (28) and Thailand (33). Frieden indicated that partnerships and coalitions with public and private sector organizations are crucial for effective implementation of public health programs (34). This study showed that the dependence on NGOs’ software programs significantly affected efforts to maintain data quality, particularly in digital health areas. Exceptional to this study finding, few key informants indicated that very hot weather conditions affected the morale of healthcare providers to take their time documenting service data on registries and tallies.

This study is not without limitations. It was qualitative, exploratory, engaging key informants that need triangulation with quantitative studies. An extensive overview of the overall barriers requires the inclusion of all health facilities, including health posts, rural health facilities, and private health facilities. The generalizability of the findings of this study may not apply to private health facilities outside of the Dire Dawa city administration. However, we believe that the barriers we found to quality of healthcare data may reflect wider systemic problems in some health facilities in Ethiopia.

## Conclusions and recommendations

This study demonstrated the complex nature of barriers to the quality of healthcare data among public health facilities in the administrative city of Dire Dawa. Multiple, some of which are mutually non-exclusive, and complex barriers to data quality were identified. The generation and maintenance of healthcare data is found to be complicated by the issues that exist throughout the process of the generation and utilization of healthcare data. Lack of standardization in all health facilities and poor commitment from management and staff were identified as bottleneck challenges in improving healthcare data quality. This study finding indicated the need for evidence-based planning and interventions on healthcare data quality from all stakeholders. Developing standardized HMIS implementation plans, delivering advanced supervisory-level training, including HMIS manuals, providing supportive supervision, and providing site-level mentorship may be very effective in identifying and resolving bottleneck data quality issues. Creating and maintaining partnerships and coalitions with public and private sector organizations is crucial. More importantly, accelerating the digitization of health information systems is very critical. Healthcare managers should understand the imperative of data quality and accept responsibility for its improvement and maintenance.

## Data Availability

The raw data supporting the conclusions of this article will be made available by the authors, without undue reservation.
